# Characterising the effect of crop species and fertilisation treatment on root fungal communities

**DOI:** 10.1038/s41598-020-74952-7

**Published:** 2020-10-30

**Authors:** Liina Soonvald, Kaire Loit, Eve Runno-Paurson, Alar Astover, Leho Tedersoo

**Affiliations:** 1 Chair of Plant Health, Institute of Agricultural and Environmental Sciences, Estonian University of Life Sciences, Kreutzwaldi 1, 51006 Tartu, Estonia; 2 Chair of Soil Science, Institute of Agricultural and Environmental Sciences, Estonian University of Life Sciences, Kreutzwaldi 1, 51006 Tartu, Estonia; 3 Chair of Crop Science and Plant Biology, Institute of Agricultural and Environmental Sciences, Estonian University of Life Sciences, Kreutzwaldi 1, 51006 Tartu, Estonia; 4 Institute of Ecology and Earth Sciences, University of Tartu, Ravila 14a, 50411 Tartu, Estonia

**Keywords:** Ecology, Microbiology, Plant sciences

## Abstract

Information about the root mycobiome may improve the overall quality of the plants and contribute to a valuable strategy to enhance sustainable agriculture. Therefore, we assessed differences in fungal community diversity and composition in the roots of potato, wheat and barley grown under mineral nitrogen fertilisation at five rates, with and without farmyard manure amendment. The same factorial combination of treatments has been used since 1989. Species richness and diversity, as well as community composition, of different fungal guilds were characterised using Illumina MiSeq sequencing of the ITS2 region. Crop species was the main factor determining overall fungal richness and diversity, with wheat showing the highest, and potato the lowest, richness and diversity. Pathogen diversity indices were highest in wheat plots amended with farmyard manure, whereas the lowest values were observed for potato roots. Fertilisation treatments and the interaction between crop species and fertilisation had the strongest impact on arbuscular mycorrhiza and saprotroph diversity. Crop species also determined the composition of the overall fungal community and that of fungal guilds, whereas fertilisation treatment had only a minor effect. This study highlights crop species as the main driver in shaping root fungal diversity and composition under the same environmental conditions.

## Introduction

The continuing challenge in agriculture is to keep increasing crop production in an environmentally sustainable manner^1,2^. In order to achieve this, one possible approach is to harness the benefits of plant-associated microbes^3,4^. Diverse plant root systems create a heterogeneous environment for microorganisms that play an important role in plant health and fitness^5^. Beneficial microorganisms improve plant nutrient uptake, liberate nutrients from organic matter and induce plant systemic resistance, whereas pathogens suppress the plant immune system and cause diseases^6^. Studying the plant–microbial interactions presents a possibility to find plant genotypes that facilitate beneficial microbial interactions, which could allow the reduction of fertiliser inputs and pesticide use^7^. However, most studies so far have focused on bacterial communities^8–11^ in spite of the importance of fungi in soil processes and plant nutrition and pathogenesis.

Wheat (*Triticum aestivum* L.) and barley (*Hordeum vulgare* L.) are among the most broadly cultivated cereals and are an important source of minerals and vitamins^12^. Potato (*Solanum tuberosum* L.) is one of the most widely grown vegetables in the world, ranking as the third most important food crop^13^. A diverse microbiome consisting of beneficial microorganisms can play an important role in sustainably increasing the yield of these economically important crops^14,15^. Studies have shown that plants may change their microbiome depending on genotype, plant root system, developmental stage and the ecosystem they inhabit^16–18^. However, we lack a comparative and comprehensive understanding of how different crops shape their microbiome.

Furthermore, we have contradictory knowledge on how different agricultural practices structure the microbiome of crops. In general, it has been shown that organic management diversifies soil microbial community composition, whereas mineral fertilisation decreases community diversity^19–21^. However, contrasting results have been reported^22,23^. Similarly, research on root fungal communities and how they respond to different fertilisation treatments has been inconsistent^24–26^. To our knowledge, only few studies have compared the root fungal community structure of different crop species under the same field environment and its response to different fertilisation practices. Wemheuer et al.^27^ determined the effect of mowing and fertilisation on endophytic fungal communities in three grassland species, in a long-term field experiment. However, they assessed the effect of management practices on fungal communities in aerial parts of plants. Hartman et al.^28^ studied the effect of fertilisation and tillage on soil- and root microbiota in a multifactor field experiment, but the study focused only on wheat.

The objective of this study was to assess differences in fungal community diversity and composition in the roots of potato, spring wheat and spring barley under different fertilisation treatments. The crops were grown in rotation under mineral nitrogen fertilisation and mineral nitrogen fertilisation combined with farmyard manure treatment. Nitrogen fertiliser was applied at five different rates. We hypothesised that crop species influence the community composition of diverse fungal guilds (pathogens, arbuscular mycorrhiza, and saprotrophs). We also tested the hypothesis that plots treated with organic manure support higher fungal richness and diversity, and reduce pathogen occurrence, compared to plots treated only with mineral nitrogen fertilisation.

## Materials and methods

### Field experiment and sample collection

The study was conducted at the field trial site located in Tartu, Estonia (58° 22.5′ N, 26° 39.8′ E). The climate here is characterised as a transitional climate zone between maritime and continental. In 2016, the mean annual temperature was 6.7 °C, and had annual rainfall of 696 mm^29^. The soil at the experimental site is classified as Fragic Glossic Retisol associated with Stagnic Luvisol (IUSS WG WRB 2015), with a sandy loam texture.

**Figure 1:**
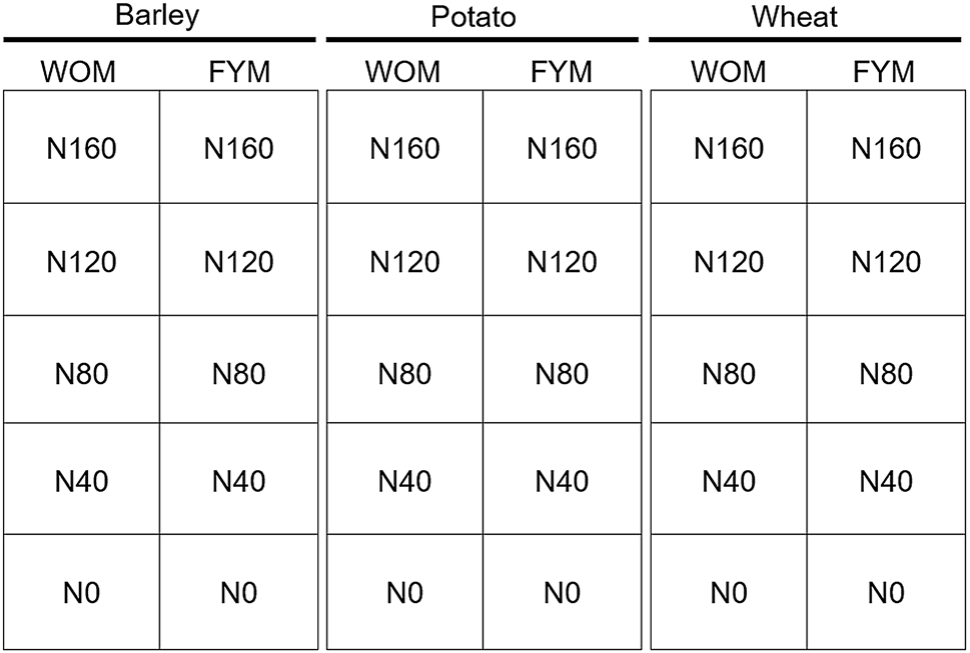
Schematic representation of one replicate block. Each block is divided into three plots planted with barley, potato and wheat, respectively. Each plot comprised two main plots treated with either mineral nitrogen fertilisation (WOM) or mineral nitrogen fertilisation combined with farmyard manure 40 t h^−1^ (FYM). The mineral nitrogen fertilisation was applied in five different application rates (N). The numbers refer to the application rate according to total N (0, 40, 80, 120, 160 kg ha^−1^).

The field experiment was arranged in a split-block design, with three replicates (Fig. 1). The treatments constituted a factorial combination of three crops, two fertilisation treatment levels and five mineral fertiliser application rates (the same crop rotation and fertilisation treatments have been used since 1989). Crops were arranged in strips across the fertilisation treatments representing the main plots, and the five nitrogen application rates as subplots (10 × 5 m). The crops studied were potato (cultivar ‘Manitou’), spring wheat (cultivar ‘Vinjett’) and spring barley (cultivar ‘Anni’). The fertilisation treatments included mineral nitrogen fertilisation (without manure, hereafter WOM) and mineral nitrogen fertilisation combined with 40 t ha^−1^ of farmyard manure (hereafter FYM). The five nitrogen fertiliser application rates were 0 (N0), 40 (N40), 80 (N80), 120 (N120) and 160 (N160) kg ha^−1^, and were applied to and mixed with the soil as ammonium nitrate during spring cultivation. Farmyard manure was applied to FYM potato plots in autumn before potato planting. An overview of treatments is provided in Supplementary Table S1. Wheat and barley root samples were collected on 20 July 2016. Due to the later planting, the potato samples were collected on 9 August 2016. Using a clean shovel, three root samples were collected from the 10–15 cm soil layer in each subplot. Each root sample consisted of the entire root system of three randomly chosen individual plants. The roots were cleaned from the soil, dried at 70 °C for 48 h, and stored dry at room temperature until molecular analysis^30^.

**Table 1:** Linear-mixed effects model examining the effect of fertilisation treatment (WOM, FYM) and fertiliser application rate (N) on soil chemical properties.

	WOM					FYM					Pr(> Chisq)^†^
	N0	N40	N80	N120	N160	N0	N40	N80	N120	N160	
pH	6.12^a^ (± 0.06)	6.02^ab^ (± 0.06)	5.96^abc^ (± 0.07)	5.76^bc^ (± 0.09)	5.68^c^ (± 0.08)	6.24 (± 0.06)	6.23 (± 0.10)	6.19 (± 0.99)	6.10 (± 0.10)	6.03 (± 0.11)	< 0.001***
C_org_	0.98 (± 0.03)	0.98 (± 0.43)	1.01 (± 0.02)	1.00 (± 0.01)	0.98 (± 0.02)	1.23 (± 0.03)	1.26 (± 0.04)	1.28 (± 0.03)	1.30 (± 0.03)	1.31 (± 0.02)	< 0.001***
N_tot_	0.06 (± 0.03)	0.07 (± 0.04)	0.07 (± 0.02)	0.07 (± 0.01)	0.07 (± 0.02)	0.09 (± 0.01)	0.09 (± 0.01)	0.11 (± 0.01)	0.09 (± 0.01)	0.10 (± 0.00)	< 0.001***
*P*	56.33^a^ (± 3.14)	47.11^ab^ (± 2.34)	44.89^b^ (± 2.29)	44.00^b^ (± 3.01)	45.89^b^ (± 2.27)	100.00^a^ (± 6.11)	93.38^ab^ (± 6.95)	85.89^b^ (± 4.53)	84.22^b^ (± 3.38)	87.00^b^ (± 3.44)	< 0.001***
K	92.22^a^ (± 5.18)	76.78^b^ (± 3.80)	77.44^b^ (± 2.96)	75.33^b^ (± 2.37)	80.22^ab^ (± 2.63)	178.56 (± 9.89)	168.25 (± 11.03)	164.78 (± 7.12)	157.22 (± 7.00)	167.44 (± 10.66)	< 0.001***

WOM, mineral nitrogen fertilisation; FYM, mineral nitrogen fertilisation combined with farmyard manure amendment; N, fertiliser application rate. The number refers to the application rate according to total N (40, 80, 120, 160 kg ha^−1^).

Values are listed as mean ± standard error. Letters indicate statistical differences between soil chemical properties within the fertilisation treatment using Tukey post hoc test following linear-mixed effects models at *P* < 0.05.

^†^Pr(> Chisq) indicates the statistical difference between fertilisation treatments.

***P < 0.001 of significance.

### Soil chemical analysis

In spring, before fertiliser application, eight subsamples, 20 cm in depth, were collected from each plot. All samples were air-dried, sieved to < 2 mm and pooled to obtain the composite sample for each plot. Soil chemical analyses were carried out to assess the amount of total nitrogen (N_total_), organic carbon (C_organic_), plant-available phosphorus (P_available_) and soil potassium (K), and the soil pH level. N_total_ was measured using the Kjeldahl method^31^ and C_organic_ was measured using the Tjurin method^32^. The ammonium lactate method^33^ was used to determine the P_available_ and K. The soil pH was determined in 1 M KCl solution.

### Molecular analysis

DNA was extracted from 75 mg of roots using the PowerSoil DNA Isolation Kit (MoBio, Carlsbad, CA, USA). We made the following modifications to the manufacturer’s protocol: 1) root samples were homogenised by bead beating with a MixerMill MM400 (Retsch, Haan, Germany) for 3 min at 30 Hz with three 3 mm autoclaved steel beads; and 2) the final elution was performed twice with 50 μl of Solution C6. PCR was performed using ITS3-Mix1-5 (CANCGATGAAGAACGYRG)^34^ and ITS3Oo (AGTATGYYTGTATCAGTGTC)^35^ forward primers and the degenerate reverse primer ITS4ngs (CCTCCSCTTATTGATATGC)^34^. The reverse primer was tagged with one of the 93 identifiers (MIDs, 10–12 bases). Each PCR mix contained 1 μl of DNA, 0.5 μl of each primer (20 pmol), 5 μl of 5xHOT FIREPol Blend Mastermix (Solis Biodyne, Tartu, Estonia) and 18 μl of PCR grade water (Solis Biodyne, Tartu, Estonia). Samples were run in duplicate on an Eppendorf Mastercycler (Hamburg, Germany) under the following conditions: initial 15 min at 95 °C, 25 cycles of 30 s at 95 °C, 30 s at 55 °C, 1 min at 72 °C, and a final cycle of 10 min at 72 °C. The products were visualised on 1% agarose gel stained with ethidium bromide to confirm successful amplification. We used PCR grade water as a negative control, and *Lentinula edodes* dry material as a positive control throughout the experiment. The duplicate PCR products were pooled, purified with a FavorPrep PCR Clean Kit (FavorGen Biotech Corporation, Vienna, Austria), and their concentrations were measured using a Qubit (Invitrogen, Life Technologies, CA, USA). Samples were sequenced on an Illumina MiSeq system (2 × 300 bp, Estonian Genome Centre, University of Tartu).

### Bioinformatics

Bioinformatic analyses were performed using the PipeCraft analysis platform^36^. The paired-end reads were quality-trimmed and assembled using vsearch v 1.1.11^37^. The resulting sequences were demultiplexed using mothur v1.36.1^38^. Chimeras were checked using de novo and reference-based (UNITE v7.2)^39^ methods as implemented in vsearch^37^. ITSx 1.0.9^40^ was used to remove flanking gene fragments and extract the full-length ITS2 region. The high-quality sequences were then clustered into operational taxonomic units (OTUs) at a 97% sequence similarity threshold with CD-Hit v4.6^41^. Singleton OTUs were removed from further analyses. For taxonomic assignment, a representative sequence from each OTU was selected for BLASTn search (word size = 11; gap open = 5; gap extension = 2; reward = 2; penalty = − 3)^42^ against the UNITE v7.2^39^ database. We conservatively considered BLASTn search results with an e-value < e−50 reliable enough to taxonomically assign OTUs. The taxonomy of OTUs was assigned based on the consensus taxonomic assignment taking into consideration the ten best BLAST hits when at least eight agreed on the same taxonomic level. The raw data of this study are publicly available through the Sequence Read Archive, BioProject PRJNA541805.

### Functional assignment

Fungal guilds of OTUs were classified using FUNGuild^43^. Where OTU fungal guild had the assignment of a plant pathogen, these were assigned as plant pathogens, (2) The guilds “plant saprotroph”, “soil saprotroph”, “dung saprotroph” and “undefined saprotroph” were merged into saprotrophic fungi. All arbuscular mycorrhizal fungi (AMF) were assigned as plant symbionts. For this study, we used the confidence rankings “probable” and “highly probable”. However, one exception was made: (1) In FunGuild, the genera *Alternaria*, *Fusarium* and *Phoma* are assigned both as plant pathogens and saprotrophs with a confidence ranking of “possible”. However, these genera are well-known soilborne fungi with split ecology^44^. Therefore, we decided also to include *Alternaria* spp., *Fusarium* spp. and *Phoma* spp. in our analysis. OTUs that were not assigned as pathogens by FUNGuild, but considered as pathogens of potato, wheat and barley, and reported in Europe according to the Agricultural Research Service of the United States Department of Agriculture (https://nt.ars-grin.gov/fungaldatabases/), were additionally assigned as pathogens. As an exception, we removed *Clonostachys* spp. from the pathogen list assigned by FUNGuild, due to its known use in agriculture as a biocontrol agent^45,46^.

### Statistical analysis

We used two ecological measures—species richness and Simpson index—to study the α-diversity of root fungal communities. Species richness was calculated based on the linear regression of OTU richness and the square root of the number of sequences to account for differences in sequencing depth^34,47^. The Simpson (1-λ) index was calculated using Primer + software on standardised and transformed tables (square-root transformation for overall fungal and arbuscular mycorrhizal abundance, and fourth-root transformation for pathogen and saprotroph abundance)^48^. A linear mixed-effects model (LMER) was used to test the effect of explanatory variables on fungal diversity indices (package “car” and “lme4” in R 3.6.0, R Development Team, https://www.R-project.org). The fixed factors included in the model were crop species, fertilisation treatment and fertiliser application rate. Replication block was included as a random factor. All tests were carried out using type II Wald Chi-Square tests. The “emmeans” package for R was used to perform the post hoc Tukey test for pairwise comparisons between variable categories. The significance threshold value was set at *P* < 0.05. In addition, LMER-analysis was applied to test the effect of fertilisation on soil chemical properties. All soil variables, except pH, were log-transformed before analysis. The model included two fixed factors (fertilisation treatment, fertiliser application rate) and one random factor (replication block). There was no significant interaction between fertilisation treatment and fertiliser application rate for any soil variable. Therefore, pairwise analysis for fertiliser application rate was conducted within the fertilisation treatment group.

As implemented in PRIMER 7 (PRIMER-E, Auckland, New Zealand), PERMANOVA + ^49^ with 9999 permutations, Monte Carlo tests, and pooling under a reduced model was used to compare the variability of fungal community composition, as well as of separate fungal guilds across experimental factors. The accompanying adjusted R^2^ value was calculated in R using the function RsquareAdj in the package “vegan”. These results were highlighted by a canonical analysis of principal coordinates (CAP)^50^. The read abundance data was standardised (by samples) and transformed (square-root transformation for overall fungal abundance, and fourth-root transformation for pathogen and saprotroph abundance) before calculating the Bray–Curtis similarity index. Due to multiple zero values in the data matrix, the analysis for AMF community composition was carried out using a modified Gower log10 resemblance matrix^51^. To test the effect of soil properties on root fungal community composition, we used the non-parametric multivariate regression DistLM^52^ in PERMANOVA + based on the abovementioned resemblance matrices. As recommended by Anderson et al.^49^, at first we looked for multicollinearity among soil properties using Draftsman plots. This led to the exclusion of K from the analysis, as it was strongly correlated with C_org_ and P_total_. Models were generated using the BEST procedure, and the best fitting model was identified using the corrected Akaike’s Information Criterion (AICc). *P* values were calculated using 9999 permutations.

A stacked bar chart was created in R using the package “ggplot”, and Venn diagrams created using the package “VennDiagram”.

## Results

### Soil properties

Fertilisation treatment and fertiliser application rate both significantly influenced soil chemical properties (Table 1). Soil pH was significantly lower in the WOM than in the FYM treatment (χ^2^ = 22.673, *P* < 0.001). Within the WOM treatment, higher fertiliser application rate significantly reduced soil pH. Soil C_organic_ (χ^2^ = 225.936, *P* < 0.001), N_total_ (χ^2^ = 59.018, *P* < 0.001), P_available_ (χ^2^ = 340.889, *P* < 0.001) and K (χ^2^ = 596.995, *P* < 0.001) were significantly higher in the FYM treatment. Furthermore, fertiliser application rate had a significant effect on soil P_available_ content within both fertilisation treatments. Within WOM and FYM treatments, N0 plots harboured significantly higher P_available_ concentration compared to N80, N120 and N160 plots. In addition, within the WOM treatment, N0 plots harboured significantly higher K content compared to N40, N80 and N120 plots.

### Identification of fungi

**Figure 2:**
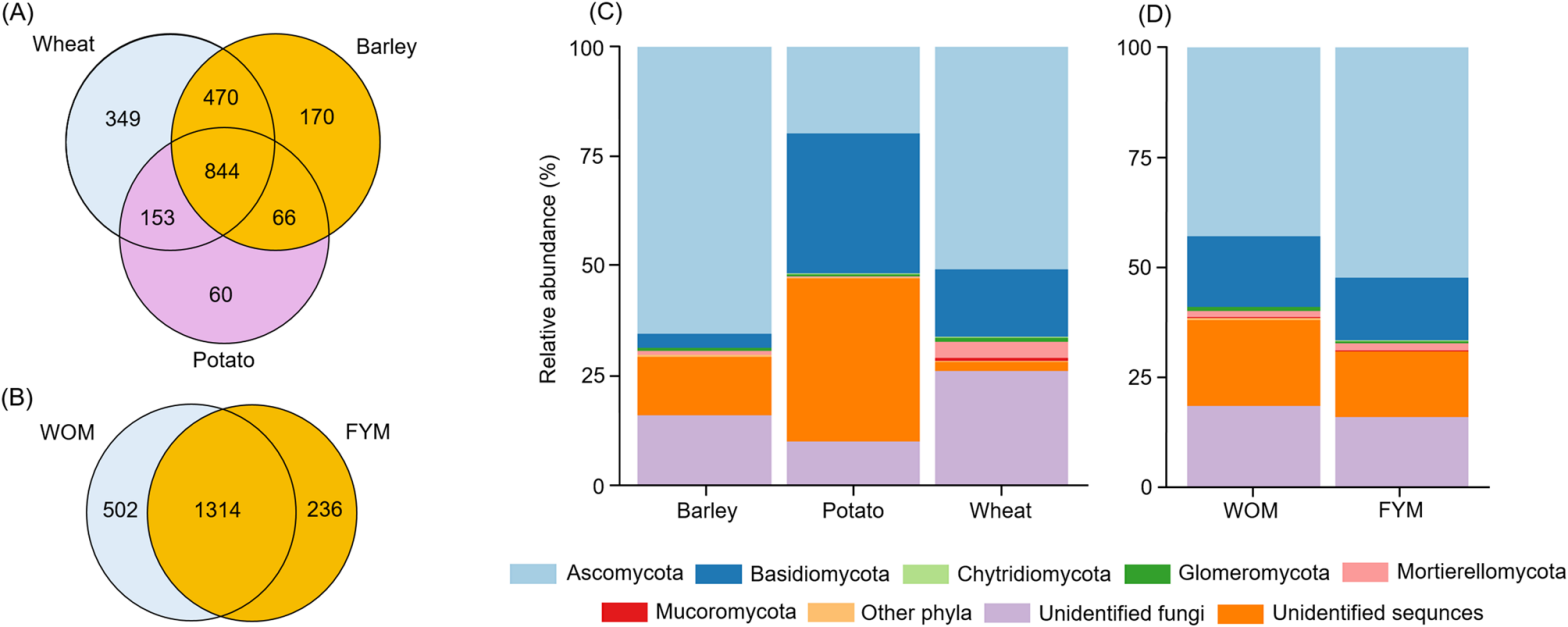
Venn diagram showing the amount of shared and unique OTUs between the roots of three crop species (**A**) and two fertilisation treatments (**B**). Taxonomic composition of root fungal communities in different crop species (**C**) and fertilisation treatments (**D**). Unidentified fungi are represented by sequences that were assigned only at kingdom level, whereas unidentified sequences represent sequences with no match. WOM, mineral nitrogen fertilisation; FYM, mineral nitrogen fertilisation combined with farmyard manure 40 t h^−1^.

Illumina sequencing of 89 samples yielded 841,519 (mean: 9455; range: 920–18,532) reads that were assigned to 2112 OTUs (Supplementary Table S2). Altogether, 844 OTUs overlapped between roots of the three studied crops (Fig. 2A), and 1514 OTUs between two of the studied fertilisation treatments (Fig. 2B). In potato roots, 37.3% of the sequences remained unidentified. Basidiomycota and Ascomycota accounted for 32.0% and 19.8% of sequences in potato roots, respectively (Fig. 2C). Ascomycota was the most abundant phylum in both wheat and barley roots, comprising 50.8% and 65.5% of sequences, respectively (Fig. 2C). Unidentified fungal sequences represented 26.0% of sequences in wheat and 15.8% in barley. The third most abundant sequences in wheat belonged to Basidiomycota with 15.5% and in barley to unidentified sequences with 13.5%. With regard to fertilisation treatment, Ascomycota was the most abundant phylum in both treatments, accounting for 43.0% and 52.5% of sequences in WOM and FYM treatments, respectively. In WOM, this was followed by unidentified sequences (19.7%) and unidentified fungi (18.3%, Fig. 2D). In FYM, this was followed by unidentified fungi (15.8%) and unidentified sequences (14.9%, Fig. 2D). Of all sequences, 27.2% were assigned to putative pathogens, 9.7% to saprotrophs, 16.6% to taxa with both pathogenic and saprotrophic features, and 0.7% to AMF symbionts. Of the ten most abundant OTUs in different crop species, at least half of these were pathogens (Table 2).

**Table 2:** Relative abundance of 10 most abundant OTUs in the roots of potato, wheat and barley.

	Potato				Wheat				Barley			
	OTU	Taxonomy	Fungal guild	%	OTU	Taxonomy	Fungal guild	%	OTU	Taxonomy	Fungal guild	%
1	Otu0536	Unidentified	Unassigned	26.8	Otu2329	*Phoma* spp.	Pathogen/Saprotroph	7.1	Otu1303	*Gaeumannomyces* spp.	Pathogen	21.9
2	Otu0930	*Rhizoctonia* spp.	Pathogen/Saprotroph	21.4	Otu1760	*Bolbitaceae* spp.	Saprotroph	5.7	Otu0164	Unidentified	Unassigned	12.9
3	Otu3047	*Colletotrichum coccodes*	Pathogen	6.2	Otu2602	*Microdochium bolleyi*	Pathogen	5.4	Otu2467	*Magnaporthaceae* spp.	Pathogen	9.6
4	Otu0364	*Thanatephorus cucumeris*	Pathogen/Saprotroph	4.7	Otu3413	*Cladosporium herbarum*	Pathogen	5.1	Otu2602	*Microdochium bolleyi*	Pathogen	5.2
5	Otu2704	Fungi	Unassigned	4.4	Otu3648	*Sordariomycetes* spp.	Unassigned	4.6	Otu3648	*Sordariomycetes* spp.	Unassigned	4.5
6	Otu0714	Unidentified	Unassigned	3.1	Otu0522	Fungi	Unassigned	3.4	Otu2329	*Phoma* spp.	Pathogen/Saprotroph	3.7
7	Otu2329	*Phoma* spp.	Pathogen/Saprotroph	2.6	Otu1363	*Exophiala equine*	Saprotroph	2.4	Otu0522	Fungi	Unassigned	3.2
8	Otu2581	*Gibellulopsis nigrescens*	Pathogen	2.3	Otu1954	Fungi	Unassigned	2.2	Otu2664	*Magnaporthaceae* spp.	Pathogen	2.5
9	Otu1943	Fungi	Unassigned	1.9	Otu1525	*Lasiosphaeriaceae* spp.	Saprotroph	2.0	Otu3413	*Cladosporium herbarum*	Pathogen	2.4
10	Otu0920	*Ceratobasidiaceae* spp.	Unassigned	1.7	Otu3288	*Fusarium* spp*.*	Pathogen/Saprotroph	1.6	Otu1525	*Lasiosphaeriaceae* spp.	Saprotroph	1.5

### Overall fungal species richness and diversity

**Figure 3:**
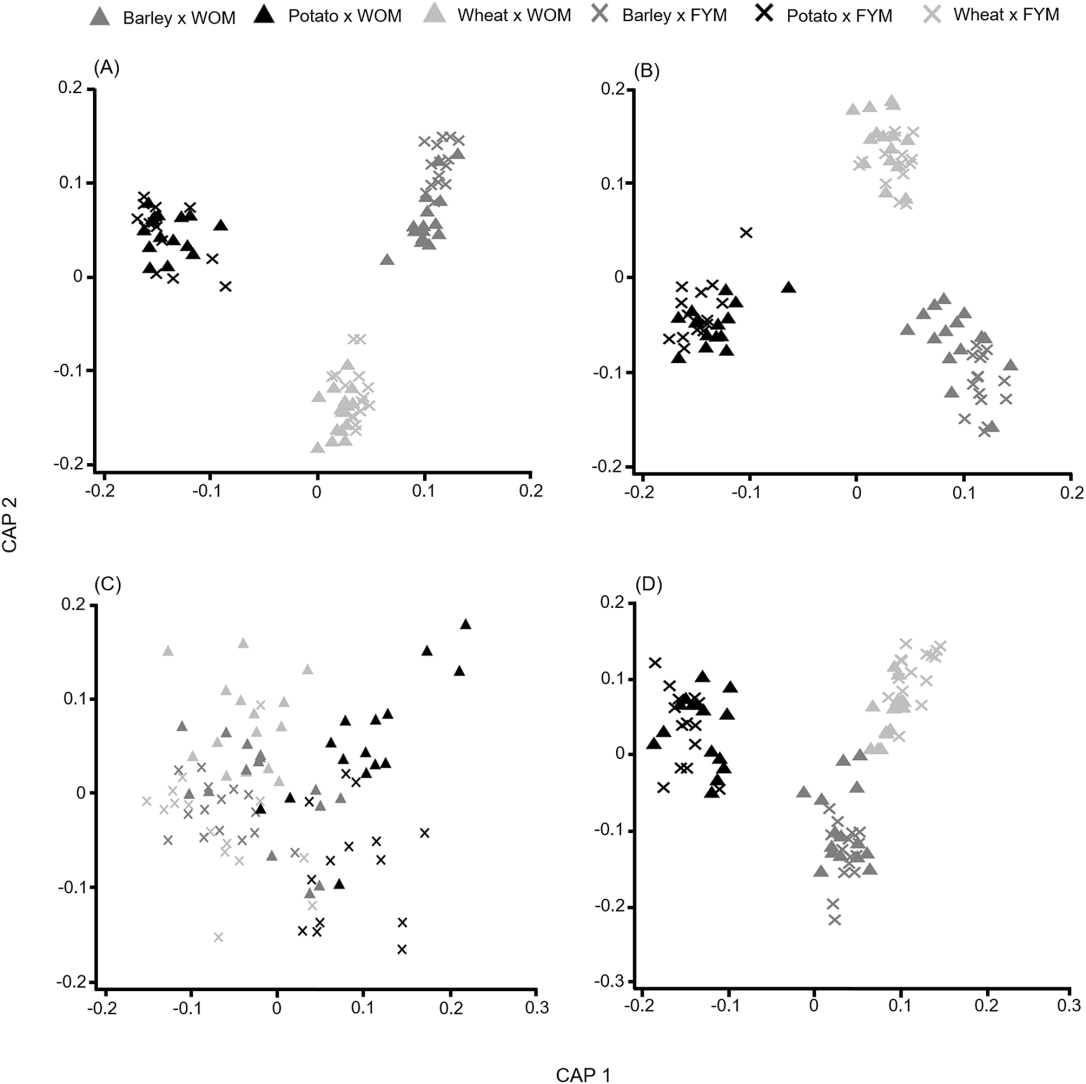
Canonical analysis of principal coordinates (CAP) based on Bray–Curtis similarity matrix (all fungi, pathogens, saprotrophs) and on modified Gower log10 matrix (arbuscular mycorrhiza) to model the effect of crop and treatment for overall fungal (**A**), pathogen (**B**), arbuscular mycorrhizal (**C**) and saprotroph (**D**) community composition. WOM, mineral nitrogen fertilisation; FYM, mineral nitrogen fertilisation combined with farmyard manure 40 t h^−1^.

Both overall fungal species richness (*P* < 0.001, Table 3) and diversity (*P* < 0.001, Table 4) differed among crop species. Species richness and diversity were highest in wheat roots and lowest in potato roots (Supplementary Table S3). PERMANOVA analysis showed that crop species (*P* < 0.001, adjusted R^2^ = 0.362) and fertilisation treatment (*P* < 0.001, adjusted R^2^ = 0.025, Supplementary Table S4) were the main factors determining the differences in fungal community composition. These results were confirmed by CAP analysis (Fig. 3A). DistLM marginal tests showed that when considered individually, each of the studied soil properties had a significant effect on fungal community composition (*P* < 0.05, Table 5). The best fitting model was achieved using the combination of pH and C_organic_, and accounted for 11.3% of the variation in the data cloud (Table 5).

**Table 3:** Results of linear mixed effect models estimating the effect of crop species, fertilisation treatment, fertiliser application rate and their interaction on species richness for all root fungi, pathogens, arbuscular mycorrhizal fungi and saprotrophs.

	*df*	Overall		Pathogens		AMF		Saprotrophs	
		Chi-Square value	Pr(> Chisq)^a^	Chi-Square value	Pr(> Chisq)	Chi-Square value	Pr(> Chisq)	Chi-Square value	Pr(> Chisq)
Crop	2	313.393	< 0.001***	209.578	< 0.001***	3.577	0.167	528.726	< 0.001***
Treatment	1	2.082	0.149	4.577	0.032*	19.374	< 0.001***	8.894	0.003**
N^b^	4	5.783	0.216	3.026	0.553	3.812	0.432	14.469	0.006**
Crop × TREATMENT	2	4.409	0.110	3.053	0.217	8.894	0.012*	8.931	0.011*
Crop × N	8	9.202	0.326	19.928	0.010*	9.723	0.285	6.086	0.638
Treatment × N	4	9.108	0.058	6.087	0.192	5.641	0.228	10.049	0.040*
Crop × treatment × N	8	13.324	0.101	7.432	0.491	11.090	0.197	14.430	0.071

*AMF* arbuscular mycorrhizal fungi, *df* degrees of freedom.

***P < 0.001 of significance; **P < 0.01 level of significance; *P < 0.05 level of significance.

^a^Pr(> Chisq) associated probability value corresponding to the test that all of the predictors are simultaneously equal to zero.

^b^N fertiliser application rate.

**Table 4:** Results of linear mixed effect models estimating the effect of crop species, fertilisation treatment, fertiliser application rate and their interaction on inverse Simpson diversity index for all root fungi, pathogens, arbuscular mycorrhizal fungi and saprotrophs.

	*df*	All fungi		Pathogens		AMF		Saprotrophs	
		Chi-Square value	Pr(> Chisq)^a^	Chi-Square value	Pr(> Chisq)	Chi-Square value	Pr(> Chisq)	Chi-Square value	Pr(> Chisq)
Crop	2	289.651	< 0.001***	148.581	< 0.001***	9.331	0.009**	253.923	< 0.001***
Treatment	1	0.220	0.639	1.182	0.277	8.194	0.004**	0.520	0.471
N^b^	4	1.872	0.759	2.946	0.567	3.802	0.433	3.099	0.541
Crop × Treatment	2	5.891	0.053	13.691	0.001**	14.951	< 0.001***	11.483	0.003**
Crop × N	8	9.858	0.275	11.419	0.179	14.988	0.059	2.745	0.949
Treatment × N	4	3.798	0.434	5.420	0.247	4.111	0.391	10.051	0.040*
Crop × Treatment × N	8	4.109	0.847	4.564	0.803	10.033	0.263	7.016	0.535

*AMF* arbuscular mycorrhizal fungi, *df* degrees of freedom.

***P < 0.001 of significance; **P < 0.01 level of significance.

^a^Pr(> Chisq) associated probability value corresponding to the test that all of the predictors are simultaneously equal to zero.

^b^N fertiliser application rate.

**Table 5:** Results of the distance-based linear model (DistLM) analysis estimating the effect of soil chemical properties for overall, pathogen, arbuscular mycorrhizal fungal and saprotroph community composition.

Marginal tests	Overall			Pathogens			AMF			Saprotrophs		
	Pseudo-F^a^	*P*^b^	Prop^c^	Pseudo-F	*P*	Prop	Pseudo-F	*P*	Prop	Pseudo-F	*P*	Prop
pH	6.278	< 0.001***	0.067	7.183	< 0.001***	0.076	2.487	0.002**	0.028	4.007	< 0.001***	0.044
C_organic_	3.454	0.002**	0.038	2.006	0.051	0.023	2.408	0.002**	0.0267	2.371	0.016*	0.027
N_total_	3.738	0.001**	0.041	3.044	0.009**	0.034	1.321	0.112	0.015	2.965	0.006**	0.033
P_available_	3.340	0.003**	0.037	1.928	0.063	0.022	3.347	< 0.001***	0.037	2.154	0.029*	0.024

	Variables	AICc^d^	R^2e^	Variables	AICc	R^2^	Variables	AICc	R^2^	Variables	AICc	R^2^
Best overall solution	pH and C_organic_	680.91	0.113	pH and N_total_	632.78	0.110	*P*	− 51.64	0.037	pH and C_organic_	652.41	0.078

AMF, arbuscular mycorrhizal fungi.

^a^Pseudo-F statistic for testing the general null hypothesis of no relation.

^b^*P* *P* value.

^c^Prop Proportion of explained variation for each variable.

^d^AICc Akaike corrected value.

^e^R^2^ Proportion of explained variation for the model.

***P < 0.001 of significance; **P < 0.01 level of significance; *P < 0.05 level of significance.

### Pathogenic fungi

*Gaeumannomyces* spp. (22.2%), *Rhizoctonia* spp. (teleomorph: *Thanatheporus* spp., 19.7%) and *Phoma* spp. (10.4%) were the most abundant pathogen genera. Crop species affected both pathogen species richness (*P* < 0.001, Table 3) and diversity (*P* < 0.001, Table 4). Both measures were highest in wheat roots and lowest in potato roots (Supplementary Table S5). Highest pathogen richness was in unfertilised (N0) wheat roots, and lowest pathogen richness in unfertilised (N0) potato roots (Supplementary Table S5). In general, pathogen richness was higher in the FYM plots (*P* = 0.003, Table 3). Furthermore, potato grown both in the FYM and WOM plots had significantly lower pathogen diversity compared both wheat and barley grown both in thein either WOM and or FYM plots (Supplementary Table S5).

Crop species was the main variable explaining the variation (*P* < 0.001, adjusted R^2^ = 0.407) in community composition, while other variables had only a minor contribution (Supplementary Table S4). Following the pattern for the total fungal community, the pathogen community composition was substantially different among all crop species (Fig. 3B). Soil pH and N_total_ were statistically significant in DistLM marginal tests, but each variable explained less than 8% of the variation (Table 5). Furthermore, the most fitting model resulted from combining pH and N_total_, and accounted for 11.1% of the variation (Table 5).

### Arbuscular mycorrhizal fungi

Of AMF, Glomeraceae was the most abundant order (81.2%), with the genus *Rhizophagus* (11.3%) dominating. AMF species richness was significantly affected by fertilisation treatment (*P* < 0.001) and crop x fertilisation treatment interaction (*P* = 0.012, Table 3). The roots of potato grown in the FYM plots showed a significant reduction in AMF richness compared to the roots of potato, barley and wheat grown in WOM plots (Supplementary Table S6). Diversity was significantly different between crop species (*P* = 0.009), fertilisation treatment (*P* = 0.004) and their interaction (*P* < 0.001, Table 4). Potato grown in FYM plots had significantly lower AMF diversity compared to any other crop and fertiliser treatment combination (Supplementary Table S6).

Crop species had a significant effect on AMF community composition, explaining 4.7% of the variation (*P* = 0.001, Supplementary Table S4). Other factors had a minor contribution to AMF community variation (Supplementary Table S4). Furthermore, CAP analysis showed only weak clustering of crop species and fertilisation treatment (Fig. 3C). DistLM marginal tests showed that pH, C_organic_ and P_available_ were significant soil properties in explaining AMF community composition (Table 5). However, the best model included only P_available_, and explained 3.7% of the total variation (Table 5).

### Saprotrophic fungi

*Rhizoctonia* spp. (32.7%), *Phoma* spp. (17.2%) and *Fusarium* spp. (9.9%) were the most abundant genera. Saprotroph species richness and diversity were significantly affected by crop species and treatment interaction (*P* < 0.001, Table 3, Table 4). Wheat grown in FYM plots harboured significantly higher saprotroph richness compared to any other crop and fertiliser treatment combination (Supplementary Table S7). Independent of crop species, saprotroph richness was the lowest in WOM plots treated with the highest fertiliser application rate (N160) (Supplementary Table S7). Saprotroph diversity was the highest in wheat and barley grown in the FYM plots, whereas the lowest values were observed in potato grown both in WOM and FYM plots (Supplementary Table S7).

PERMANOVA analysis showed a significant effect of crop species (*P* < 0.001, adjusted R^2^ = 0.275) and fertilisation treatment (*P* = 0.007, adjusted R^2^ = 0.012) on saprotroph community composition (Supplementary Table S4). According to CAP analysis, considerably different saprotroph community compositions were observed depending on both crop species and fertilisation treatment (Fig. 3D). DistLM analysis showed a significant effect of each soil variable on saprotroph community composition in marginal tests (Table 5). However, each variable explained less than 5% of the variation. Moreover, the best model included only pH and C_organic_ as predictors and explained 7.8% of the total variation (Table 5).

## Discussion

We documented the patterns of root fungal communities in response to three crop species, two types of fertilisation treatment and five fertiliser application rates. In support of our first hypothesis, fungal community diversity and composition differed substantially among crop species, indicating that agricultural plant species shape their root mycobiome. How plants affect their fungal communities can be related to differences in root traits and root exudates^53,54^. Plants produce root exudates that vary between plant species and thus establish a unique root microbe community^55^. These differences are more significant between phylogenetically distant species^56^.

Moreover, roots also secrete root border cells and mucilage, both of which can vary between plant species^57,58^. Koroney et al.^59^ showed that there are galactan-containing polymers in potato mucilage. In wheat roots, the abundance of galactan-containing polymers has been observed to be relatively low^60^; thus galactan-containing polymer content may be one cause for the differences in root microbe communities between cereals and potato, observed in our study. In addition, plant root architecture can influence microbial communities both directly and indirectly^5^. Both overall fungal- and pathogen diversity were greatest in wheat, followed by barley and potato. The higher fungal diversity in cereals, compared with potato, may be related to their more differentiated root structure^61^ or phylogenetic effects^62^. Cereals have strong fibrous root systems, which branch throughout the life of the plant^63–65^, whereas the potato root system is considered shallow and sparse^66^. Furthermore, wheat plants exhibit a higher total volume of roots, compared to potato^67^. Therefore, the greater root surface area of cereals may provide more adhesion sites for fungi.

Our study revealed a relatively high frequency of pathogens compared with previous studies in agricultural fields^68^ and forests^69^. The particularly high abundance of pathogens on barley may be related to crop rotation. The most abundant OTU in barley was identified as *Gaeumannomyces* spp., which are common root disease agents in various cereals. In our study, barley followed wheat in crop rotation. Having suitable plant hosts in rotation across two consecutive years may have allowed the accumulation of pathogens. These results are consistent with Chen et al.^70^ and Song et al.^71^, who showed the effect of continuous cropping on pathogen increase. Different crop species in the rotation that do not share common pathogens can help to break the life cycle of plant pathogens and hinder their establishment in the field over time^72^.

Root symbiotic AMF accounted for < 1% of sequences, which is in accordance with previous studies showing a low amount of Glomeromycota rRNA genes in the roots of crop plants^73^. While the AMF assemblages were similar between wheat and barley, potato showed greater differences. Plant host could be the major determinant affecting root AMF communities^74^, but this may also be related to differences in root structure, phylogenetic distance, or our three-week interval between sampling events.

Saprotrophs also showed distinct communities in roots of crop species. In line with this study, Francioli et al.^75^ have shown plant species is the main factor in shaping the root-associated saprophytic fungal community. They argued that the variation between communities may be driven by differences in C:N ratio and root lignin content. Furthermore, Mariotte et al.^76^ highlighted the importance of different organic inputs in decomposer communities. Therefore, saprotrophs may have developed plant tissue specificity, allowing the development of distinct saprotroph communities in the crop roots. It is also possible that some of these saprotrophs act as pathogens in certain plant species or fertilisation treatments, which may favour their accumulation in specific plant taxa.

The total fungal community showed no response to fertilisation treatments. In previous studies, both inorganic and organic nitrogen fertilisation have demonstrated substantial effects on fungal diversity and composition in agricultural plants^77,78^. Furthermore, in this study soil, chemical properties were significantly different between WOM and FYM plots. However, soil properties had a relatively weak effect in determining fungal community composition. This may be related to a lower fungal sensitivity towards changes in soil properties^79–81^. It is possible that after several years of fertilisation at our field site, the local fungal communities had been selected to tolerate high levels of fertilisation and continuous disturbance (tillage), and therefore, here, fertilisation type and application rate play minor roles in shaping the root fungal microbiome. A stable fungal community in response to long-term fertiliser amendment has also been observed by Marschner et al.^82^ and Ai et al.^83^.

Pathogen richness and diversity were higher in the roots of wheat and barley grown in FYM plots. Farmyard manure amendments may result in a more eutrophic environment^84,85^ and, together with the more complex root structure of wheat and barley provide a more suitable habitat for pathogens. This assumption is supported by the fact that potato roots harboured the lowest pathogen richness and diversity.

In general, the lowest AMF diversity was observed in FYM plots, whereas soil nutrient levels, including P_available,_ were highest in FYM plots. Studies have shown that higher phosphorus concentration can decrease AMF colonisation in roots and may cause a shift in soil AMF community composition^86,87^. It is possible that in WOM plots, AMF mediated nutrient acquisition for the crops. However, in FYM plots, the manure amendment may have saturated soil nutrient concentrations, reducing AMF diversity. This assumption is supported by the DistLM analysis, which suggested that P_available_ is the only soil variable influencing AMF community composition. Nutrient saturation may also explain the lowest AMF richness and diversity in potato grown in FYM plots since these plots had the most recent farmyard manure amendment.

Saprotrophs were generally more diverse in FYM plots. Results showing an increase in saprotroph diversity in manure-amendment-treated fields have also been reported in other recent studies^88,89^. Saprotrophs are important for decomposing and mineralising organic matter in agricultural soils^44,90^, and thus a positive relationship between soil organic matter and saprotroph richness and diversity may be expected. The three most abundant saprotroph taxa (*Rhizoctonia* spp*.*, *Phoma* spp*.*, *Fusarium* spp*.*) were also assigned as pathogens. Members of these genera are common soil inhabitants that become pathogenic under favourable conditions^44^. We speculate that although manure amendment itself did not affect pathogen communities in our study, its beneficial impact relies on the increase in fungi with saprotrophic characteristics. This is in agreement with earlier observations that several plant pathogens are viable on organic matter and increase their inocula due to a saprotrophic mode of nutrition^91,92^.

## Conclusion

Root fungal diversity and composition are strongly shaped by crop species, the effect of which prevails over that of fertilisation treatment. The relatively small effect of fertilisation treatment and fertiliser application rate may be explained by the stability of the local agricultural system after years of fertilisation. Therefore, our results indicate that within a conventional system, organic manure amendment does not enhance the root mycobiome. Although the root mycobiome remained relatively unaffected by fertilisation treatment, nitrogen fertilisation may affect bacteria, free-living soil fungi or soil conditions. To gain further insights into the interactions between agricultural management and microbiomes, future studies should be carried out on multi-crop experimental sites, at larger spatial scales, and include additional groups of microorganisms.

## Supplementary information

Supplementary Tables.
